# Exploring the Manifestation of Non-motor Symptoms in Parkinson's Disease in a Tertiary Care Center: A Comprehensive Analysis

**DOI:** 10.7759/cureus.87633

**Published:** 2025-07-09

**Authors:** Deepak S Gonaboyina, Pragateshnu Das, Nikhilesh Pradhan, Prerana Dash, Vipin Venugopalan, Santosh Dash

**Affiliations:** 1 Department of Neurology, Kalinga Institute of Medical Sciences, Bhubaneswar, IND

**Keywords:** eastern india, gastrointestinal symptoms, mds-nms scale, non-motor symptom, parkinson' s disease

## Abstract

Background: Non-motor symptoms (NMS) in Parkinson’s disease (PD) can influence cognition, emotion, sleep, and autonomic systems. NMS frequently precede motor symptoms, underscoring the necessity for a thorough comprehension of these symptoms, especially in heterogeneous populations like India. This study aims to explore the NMS of PD in a cohort from eastern India.

Materials and methods: Sixty consecutive patients with PD were enrolled in this study after considering the inclusion and exclusion criteria. Informed consent was obtained from each patient after ethical clearance from the institutional ethics committee. Data were collected by using the standardized Movement Disorder Society Non-Motor Symptoms Rating Scale (MDS-NMS) to assess various NMS. Additionally, the study categorized patients into clinical subtypes based on their predominant symptoms and it analyzed how NMS manifested in each subtype. The correlation between NMS was also assessed.

Result: Gastrointestinal (GI) issues were the most common NMS, affecting all 60 (100%) patients. Depression affected 59 (98.33%) patients, followed by anxiety in 57 (95%) patients. Only two (3.33%) patients reported sexual dysfunction, while 55 (91.67%) patients reported pain, and 54 (90%) patients reported cognitive impairment. Constipation was more common among symptoms of the GI domain. Twenty-seven (45%) patients had developed at least one NMS prior to the diagnosis of PD. Younger patients (<60 years) tend to experience more severe emotional symptoms like depression and anxiety. Older patients (60-80 years) showed increasing severity of physical symptoms like pain, fatigue, and GI issues, along with cognitive decline. No statistically significant differences were observed for NMS severity between the akinetic-rigid versus tremor-predominant subtypes of PD.

Conclusion: The findings emphasize that NMS are prevalent in nearly all PD patients. Addressing NMS in clinical practice is crucial for improving patient outcomes. Particularly in India, where these symptoms may be underdiagnosed, the study highlights the need for routine screening and management of NMS to enhance overall care strategies for PD patients.

## Introduction

Parkinson’s disease (PD) is the second most prevalent neurodegenerative disorder worldwide, following Alzheimer's disease [1]. It predominantly affects older adults, with prevalence increasing significantly with age. Traditionally, PD research and treatment have focused on motor symptoms such as tremors, bradykinesia, and rigidity resulting from depletion of dopamine in the brain. However, recent studies underscore the significant impact of non-motor symptoms (NMS), which can affect cognition, mood, sleep, and autonomic functions [2]. NMS often precedes motor symptoms, highlighting the need for a comprehensive understanding of these symptoms, particularly in diverse populations such as India, where research is limited [3]. In the Indian context, studies indicate a high prevalence of NMS among PD patients, ranging from 93.75% to 100% [4,5]. Despite their frequency, NMS remain frequently underdiagnosed, overlooked, or inadequately managed, emphasizing the need for improved diagnostic tools, systematic screening methods, and targeted therapeutic strategies tailored specifically to address NMS among PD patients. Timely recognition and effective management of these symptoms can substantially enhance patients' quality of life and overall clinical outcomes [6]. The purpose of the study was to determine the type, frequency, and severity of NMS in PD patients and to analyze NMS distribution and association with clinical subtypes of PD, ultimately guiding better clinical practice. 

## Materials and methods

Study design

This was a cross-sectional observational study designed to evaluate the frequency, type, and severity of NMS in patients diagnosed with PD.

Study setting and duration

The study was conducted at the Department of Neurology, Kalinga Institute of Medical Sciences (KIMS), Bhubaneswar, Odisha, India, from October 2022 to August 2023.

Participants

Sixty consecutive cases satisfying our inclusion and exclusion criteria were enrolled.

Inclusion criteria

All consecutive patients included in the study had a confirmed diagnosis of PD based on the Revised International Parkinson and Movement Disorder Society clinical diagnostic criteria [7]. Only those individuals who demonstrated the willingness and ability to provide informed consent, as well as the capacity to participate in interviews and complete clinical evaluations, were considered eligible for inclusion.

Exclusion criteria

Individuals with a Montreal Cognitive Assessment (MoCA) score below 18 were excluded in order to minimize the influence of cognitive impairment on the evaluation of NMS. Patients with disabling concomitant neurological disorders such as stroke, severe head trauma, or other neurodegenerative diseases were also not considered for inclusion. Furthermore, individuals with significant comorbidities, including active oncological conditions, major cardiac illnesses, chronic obstructive pulmonary disease, end-stage liver disease, or end-stage renal disease, were excluded. Patients experiencing active neuropsychiatric disturbances that could impair their ability to provide informed consent or meaningfully participate in assessments were likewise not included in the study.

Ethical consideration

This study was started following ethical clearance by the Institutional Ethics Committee, Kalinga Institute of Medical Sciences, Bhubaneswar (IEC number: KIIT/KIMS/IEC/1063/2022). Informed written consent was obtained from all the enrolled patients.

Data collection and outcome measures

Data pertaining to the socio-demographic characteristics and clinical profile were collected. PD was evaluated according to the Movement Disorder Society Unified Parkinson's Disease Rating Scale III (MDS-UPDRS scale III) [8]. The clinical type at the onset of the disease was classified as tremor dominant and akinetic-rigid. Various NMS were assessed via clinical interview and examination. The Movement Disorder Society Non-Motor Symptoms Rating Scale (MDS-NMS) was used to evaluate NMS after taking the required permission from the International Parkinson and Movement Disorder Society.

Statistical analysis

Demographic and clinical variables were analyzed using both parametric and non-parametric tests, depending on the data distribution, with Statistical Package for Social Sciences (SPSS) software version 26.0 (IBM Corp., Armonk, NY). Quantitative data were presented as mean ± standard deviation (SD) or median and interquartile range, while categorical variables were expressed as numbers and percentages. Descriptive statistics were employed to determine the prevalence of disease in the patient cohort. Pearson’s correlation matrix was utilized to examine the relationships between various NMS. The comparison of NMS prevalence between clinical phenotypes, such as tremor dominant and akinetic-rigid, was conducted using ANOVA to assess statistical significance between groups.

## Results

The study was conducted with a sample of 18 females (30%) and 42 males (70%). The average age for the female participants was recorded as 63 ± 8.15 years. In contrast, the male participants had a slightly higher average age of 68.9 ± 6.16 years. The average age of disease onset was 62.9 ± 6.8 years. The mean duration of the disease at the time of the study was 4.25 ± 1.6 years. The side of onset data showed that 33 patients (55%) experienced the disease onset on the right side, and 27 patients (45%) experienced the onset on the left side. Tremor predominant was the most common type, affecting 40 patients (66.6%). Akinetic-rigid type affects 20 patients (33.3%) (Table 1).

**Table 1:** Demographic data of participants: age and gender distribution; age at disease onset and disease duration; side of disease onset and clinical type

Demographic Data	Value
Total participants (n)	60
Gender distribution, n (%)	
Male: female ratio	42:18 (70%:30%)
Age distribution (mean ± STD)	
Mean age in years	67.1 ± 7.28
Mean age (males)	68.9 ± 6.16
Mean age (females)	63.0 ± 8.15
Mean age of disease onset (years)	62.9 ± 6.8
Mean disease duration (years)	4.25 ± 1.6
Side of disease onset, n (%)	
Right	33 (55%)
Left	27 (45%)
Clinical subtype, n (%)	
Tremor predominant	40 (66.6%)
Akinetic-rigid	20 (33.3%)

**Figure 1:**
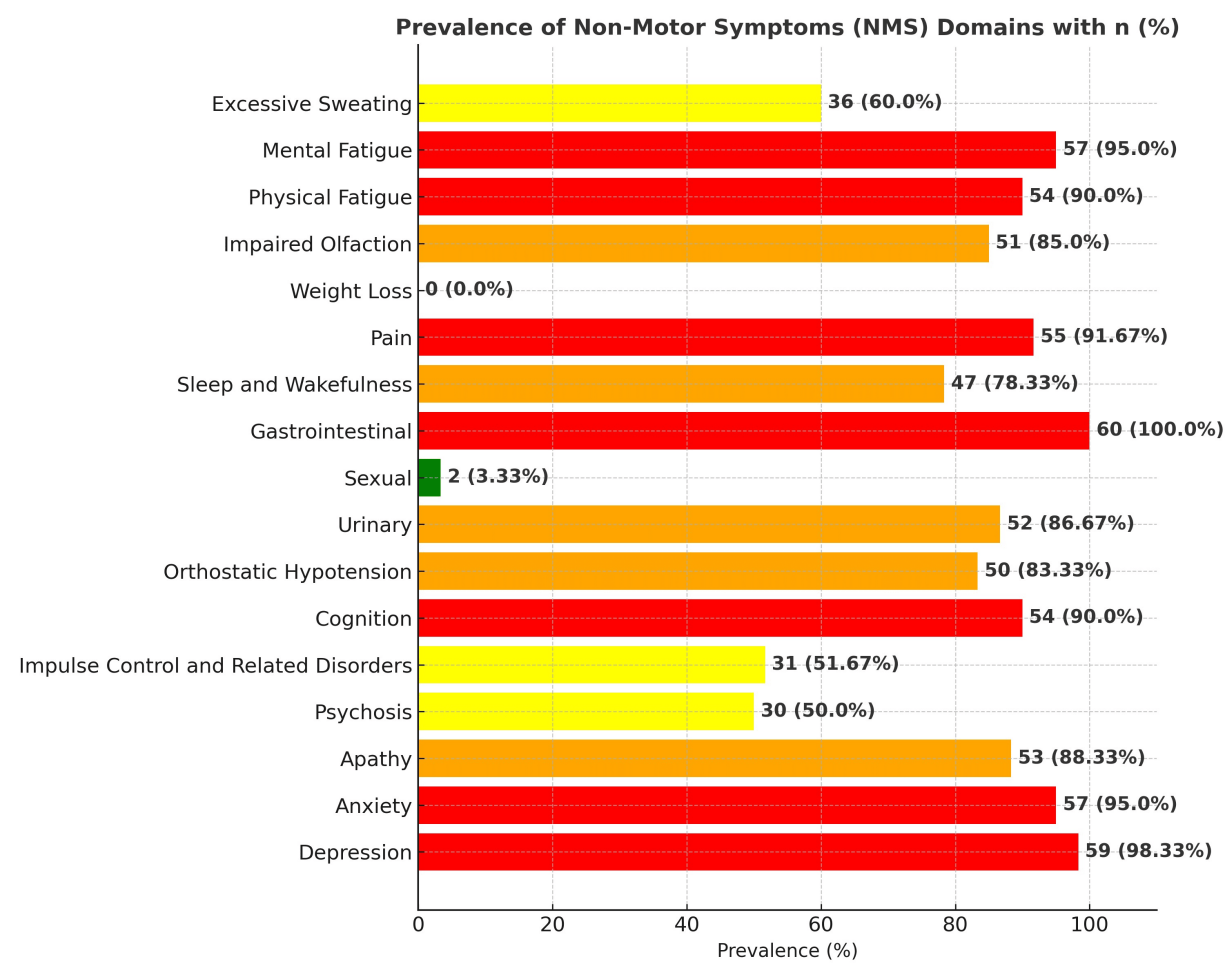
Prevalence of NMS domains. GI symptoms, depression, and anxiety were the most prevalent in this study population NMS, non-motor symptom; GI, gastrointestinal

In our study, depression was highly prevalent, affecting 59 patients (98.3%), while anxiety was present in 57 patients (95%) of the cohort. Apathy, psychosis, impulse control-related disorders, and cognitive issues were reported in 53 patients (88.33%), 30 patients (50%), 31 patients (51.67%), and 54 patients (90%), respectively. Orthostatic hypotension was found in 50 patients (83.33%), and urinary problems were reported by 52 patients (86.67%). However, sexual dysfunction was relatively uncommon, affecting only two patients (3.33%). Interestingly, gastrointestinal (GI) issues were present in all 60 patients (100%), making it the most universal condition. Sleep and wakefulness issues affected 47 patients (78.33%) of the cohort, while pain was reported by 55 patients (91.67%). Weight loss, however, was not reported by any patients. Impaired olfaction was prevalent in 51 patients (85%), and both physical and mental fatigue affected 54 patients (90%) and 57 patients (95%), respectively. Finally, excessive sweating was noted in 36 patients (60%). This indicated that a high prevalence of various NMS, with GI issues, depression, and anxiety, was particularly common among the patient population (Figure 1).

**Figure 2:**
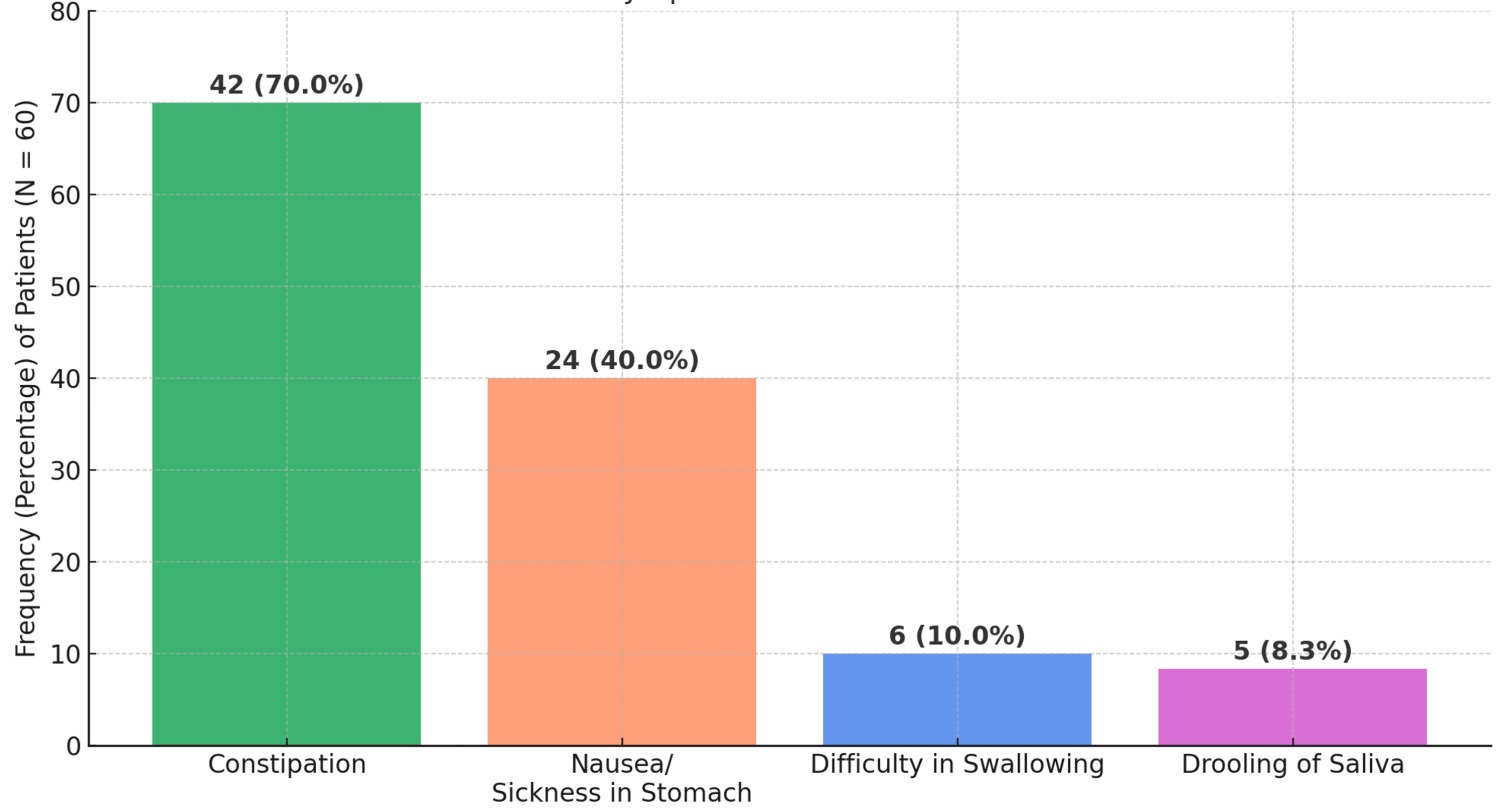
Occurrence of individual symptoms in the GI domain Due to the overlap and co-occurrence of symptoms, such as constipation with nausea or difficulty swallowing with excessive salivation, the sum of percentages exceeded 100%. Consequently, the total number of symptom reports was higher than the number of patients (n=60), as individual patients frequently experienced multiple GI symptoms simultaneously. GI, gastrointestinal

All patients in our study cohort reported at least one symptom within the GI domain; hence, the prevalence of specific GI-related symptoms was analyzed further. The findings showed 42 patients (70%) reported constipation. Twenty-four patients (40%) experienced nausea or stomach sickness. Six patients (10%) had difficulty swallowing (dysphagia), and five patients (8.33%) complained of excessive drooling of saliva (sialorrhea). This highlighted a high burden of GI dysfunction among the patients, with constipation being the most frequently reported symptom (Figure 2).

On further analysis, 27 (45%) out of 60 patients had developed at least one NMS prior to the diagnosis of PD. Twenty-three patients (38.3%) experienced only one symptom; four patients (6.67%) experienced two symptoms simultaneously. Constipation coexisted with both anosmia/hyposmia and restless leg syndrome (RLS) in different subsets of patients.

**Figure 3:**
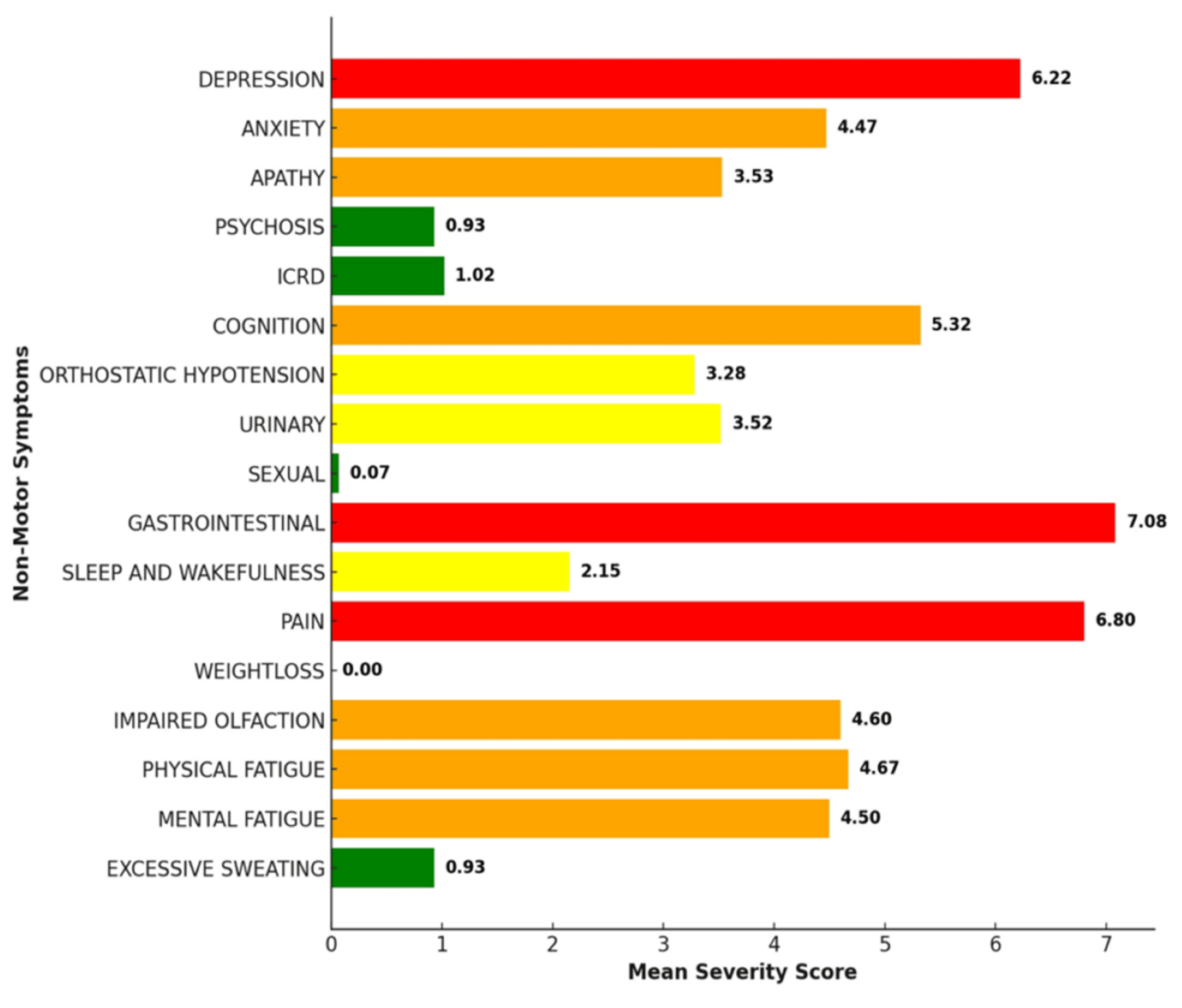
Mean severity scores of individual NMS domain GI symptoms and pain followed by depression were more severe and burdensome in the patient cohort. NMS, non-motor symptom; GI, gastrointestinal

The severity of each NMS contributing to the overall NMS burden was assessed by the mean severity score for each symptom. By averaging the scores of each symptom's overall severity, the burden was calculated. Mean severity scores are the sum of all severity scores for the symptom divided by the number of patients. The severity analysis of NMS in the patient cohort revealed that depression had a mean severity score of 6.22, indicating a relatively high level of impact, while anxiety was slightly lower with a mean score of 4.47. Apathy followed with a score of 3.53. In contrast, psychosis had a notably low mean severity score of 0.93. Similarly, impulse control and related disorders were also low, with a mean score of 1.02. Cognition issues were moderately severe, with a mean score of 5.32, while orthostatic hypotension and urinary problems scored 3.28 and 3.52, respectively. Sexual dysfunction had a very low mean severity score of 0.07. GI issues, however, stood out as the most severe, with a mean score of 7.08. Sleep and wakefulness issues had a mean score of 2.15, while pain was also significant with a score of 6.80. Weight loss showed no reported severity (0.00), and impaired olfaction had a mean score of 4.60. Both physical fatigue and mental fatigue were reported with moderate severity, scoring 4.67 and 4.50, respectively. Finally, excessive sweating, like psychosis, had a low mean severity score of 0.93. These results showed that GI issues, pain, and depression were among the most severe NMS, while sexual dysfunction and psychosis were reported with low severity (Figure 3).

**Figure 4:**
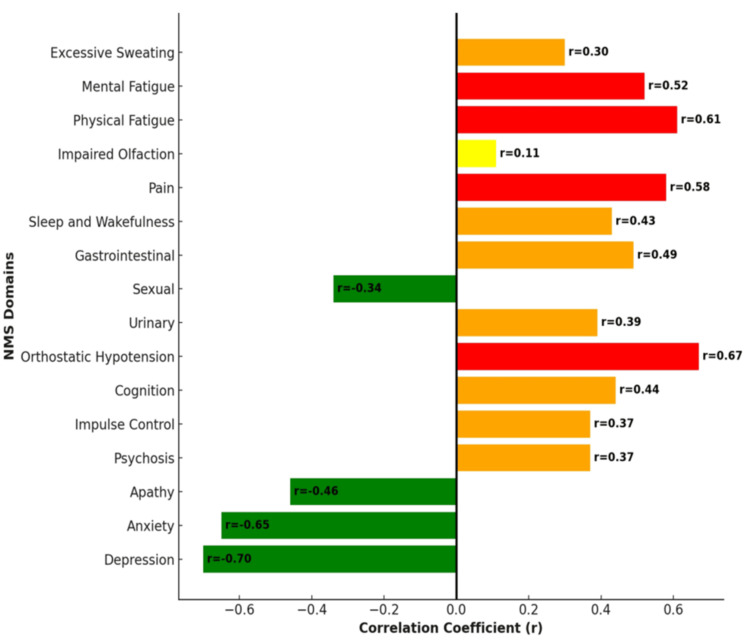
Correlation between age and symptom severity Younger patients exhibited more emotional disturbances, while older patients experienced more physical disturbances such as pain and GI symptoms. NMS, non-motor symptom; GI, gastrointestinal

**Figure 5:**
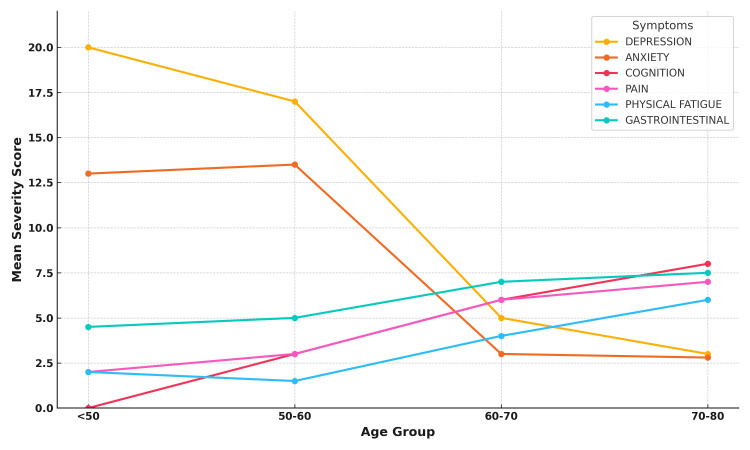
Age-specific symptom severity trend Emotional symptoms were more common in the younger population, while the severity of physical symptoms tended to increase with age.

The correlation between age and the severity of each NMS domain revealed that orthostatic hypotension, pain, and physical fatigue had a positive correlation with age, indicating that their severity tended to increase with advancing age. Conversely, depression and anxiety showed a strong negative correlation, indicating their severity decreased with age (Figure 4). Younger patients (<60 years) tend to experience more severe emotional symptoms like depression and anxiety. Older patients (60-80 years) showed increasing severity of physical symptoms, including pain, GI issues, physical fatigue, and cognitive decline. This graph (Figure 5) below highlights the importance of focusing on various types of NMS based on the patient's age, with younger patients potentially needing more support for emotional health and older patients requiring management of physical and cognitive decline.

**Figure 6:**
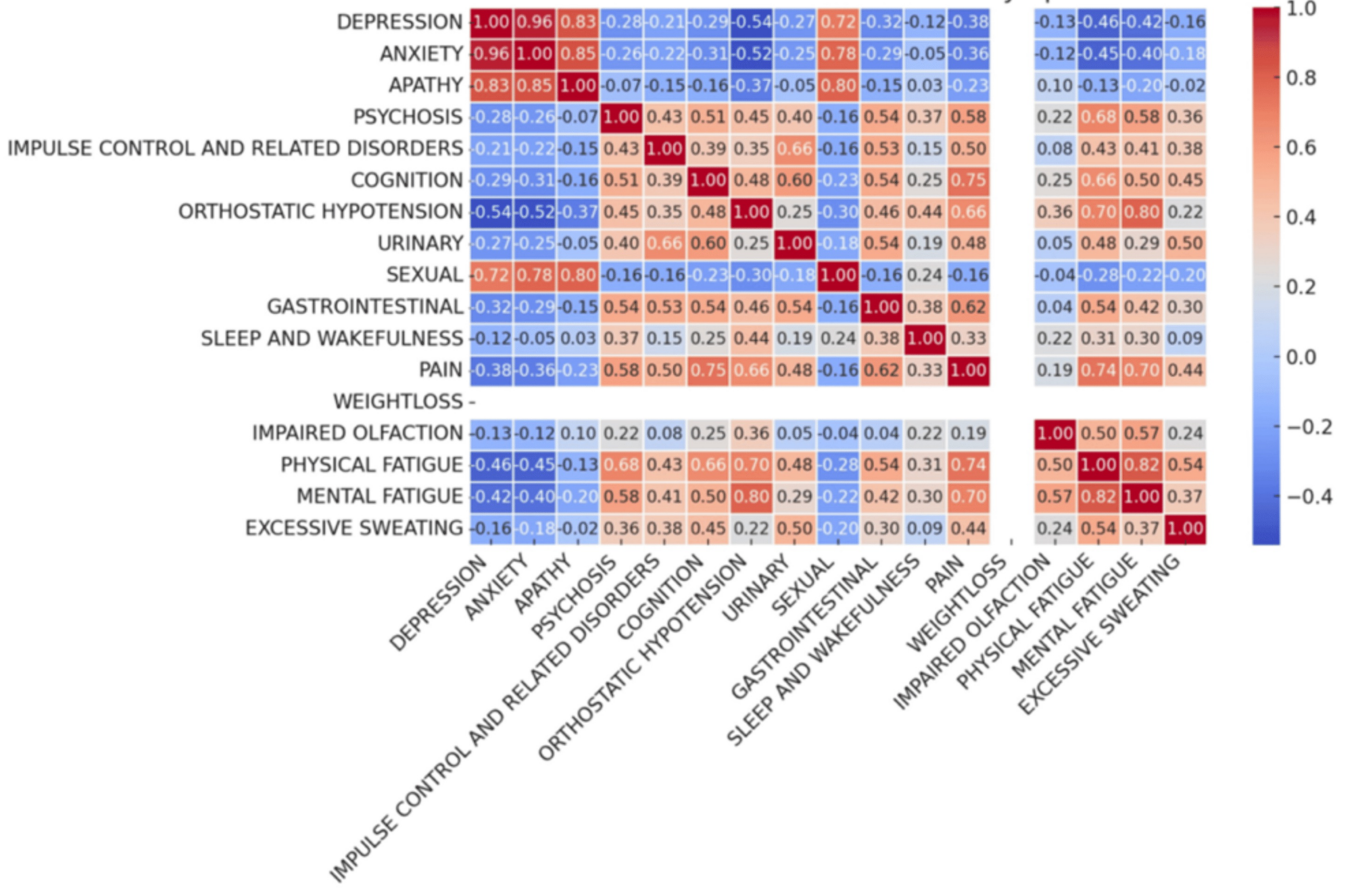
Correlation matrix among the NMS domains Apart from the strong correlation of emotional symptoms, pain is moderately correlated with cognition and fatigue. NMS, non-motor symptom

The correlation matrix (Figure 6) among the NMS indicated the relationships between different symptoms, with values ranging from -1 to 1. Positive values indicated a direct correlation, meaning that as one symptom's severity increases, the other tends to increase as well. Negative values indicate an inverse relationship. Very strong correlations were observed among emotional symptoms such as depression, anxiety, and apathy, indicating that these mood-related issues tended to be co-occurring. Pain was identified as central to several symptom relationships, particularly with fatigue and cognitive issues, reflecting its far-reaching impact on patient well-being. Additionally, increases in pain, physical fatigue, and GI issues were observed with longer disease duration.

**Figure 7:**
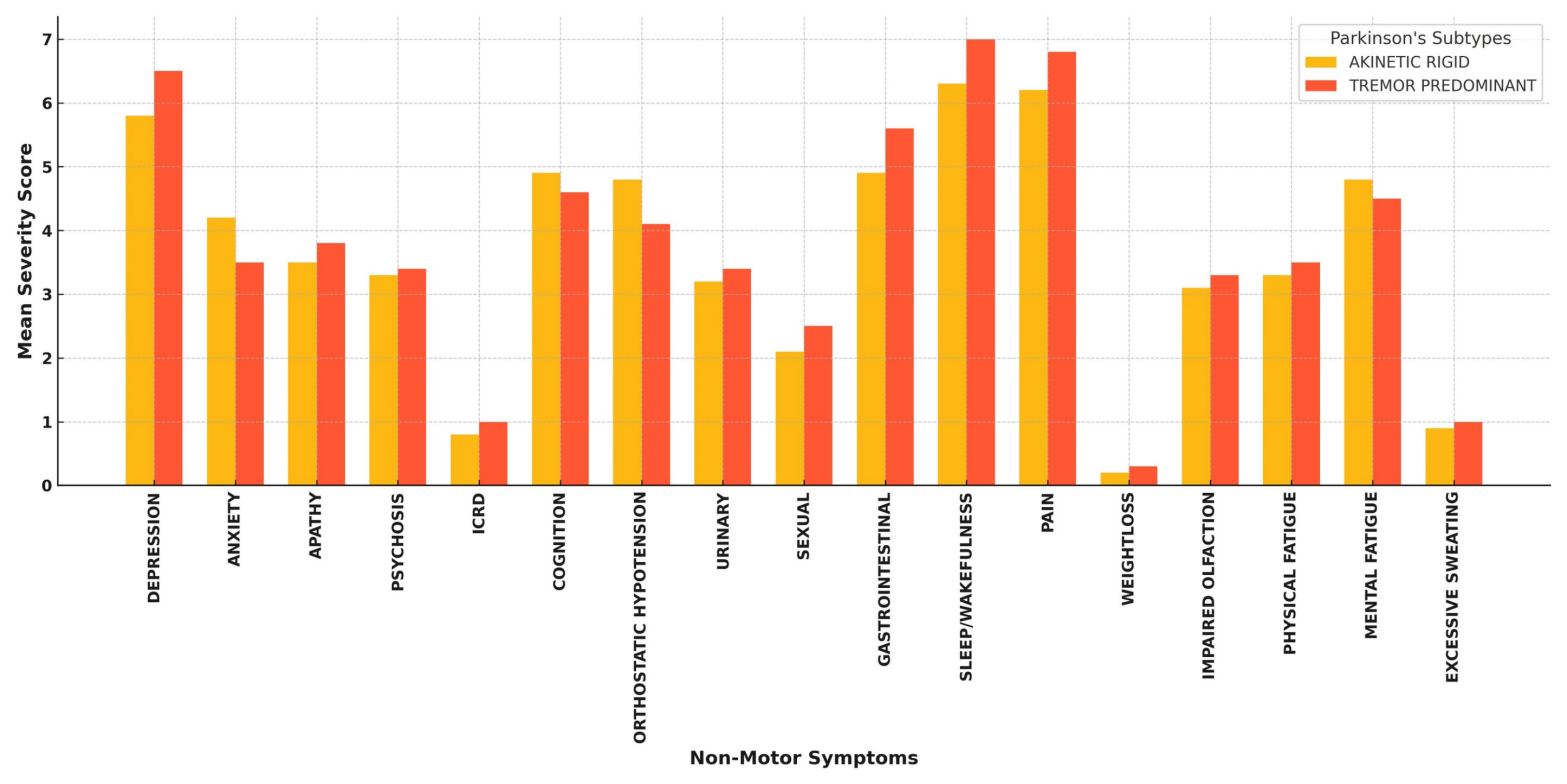
Incidence of individual NMS domains among PD subtypes PD, Parkinson's disease; NMS, non-motor symptom

Further analysis of mean severity scores for individual NMS domains among PD subtypes revealed that the tremor-predominant group exhibited a higher prevalence of depression, pain, and cognitive issues, while the akinetic-rigid group showed increased incidences of GI symptoms and orthostatic hypotension. However, no statistically significant differences were observed between the akinetic-rigid versus tremor-predominant subtypes of PD (Figure 7).

## Discussion

The demographic profile of our study, which included 60 PD patients, was male predominant (M:F = 7:3), with a mean age of 67.1 ± 7.28 years. This profile aligned with the well-documented epidemiology of PD. The disease has been reported to be more common in men, as confirmed by Pringsheim et al., who found that PD is approximately 1.5 times more prevalent in men than in women [9]. Additionally, the age distribution observed in our study was consistent with data from the Global Burden of Disease Study (2016), which indicated that PD primarily affects individuals over 60 years of age [10]. The standard deviation of age (7.28 years) was found to suggest variability, which was seen to correspond with the findings of Titova et al., who also noted that age had been shown to significantly impact both motor and NMS in PD patients [11].

There is statistical significance in the number of cases (p = 0.00157) and age of presentation (p = 0.00315) in the study population cohort. Such a pronounced gender imbalance suggests that males may be more prone to seeking medical care or have a higher prevalence of the condition being studied, aligning with findings from other studies in similar socio-cultural settings. In the context of PD in India, several studies have highlighted significant gender disparities in both prevalence and disease presentation. Males had a higher prevalence of PD and tended to present at a later age compared to females. This study suggested that cultural and social factors might influence these gender differences, with men being more likely to seek medical care or face different environmental risk factors. Similarly, Behari et al. noted that differences in healthcare access between genders could further exacerbate these disparities, reflecting broader issues within the Indian healthcare system [12]. These findings underscore the need to account for gender-specific factors in the diagnosis and management of PD in India.

In our study, the average age of disease onset was found to be 63 ± 6.8 years, with an average disease duration of 4.25 years. This was observed to be consistent with the findings of Kalia and Lang, who documented that PD had typically been manifested in the early 60s [13]. Moreover, the variability in onset age (46-74 years) and the progression of the disease observed in our study were found to be reflective of the progressive nature of PD, as had been reported by Hely et al. [14].

In our cohort, 45% of patients developed at least one NMS before a PD diagnosis, with constipation being the most frequent, occurring on average 5.07 years prior to diagnosis. Indian research supports the early onset of constipation, often up to 10 years before motor symptoms, highlighting its role as a potential early diagnostic marker [15]. Anosmia/hyposmia was reported in 12 patients, with a mean duration of 1.59 years before diagnosis. Olfactory dysfunction is a well-established early indicator of PD, and multiple studies recommend its inclusion in screening for PD [16]. RLS was less common, reported by five patients with an average onset of 0.30 years before diagnosis. RLS may share mechanisms with other NMS like constipation or anosmia, though more Indian research is needed to fully understand its prevalence and impact on PD diagnosis.

The results were found to indicate a high prevalence of NMS, with GI issues in 60 patients (100%), depression in 59 patients (98.33%), and anxiety in 57 patients (95%) being particularly prominent. This was observed to align with studies such as Sauerbier et al., which had reported similar findings regarding the prevalence of NMS in PD patients [17]. Poewe et al. also highlighted that NMS were often under-recognized but were found to have a significant impact on the quality of life of PD patients [18].

The high prevalence of depression (98.33%) was found to be consistent with findings from Aarsland et al., who had observed that up to 90% of PD patients experience depression at some stage of the disease [19]. Similarly, anxiety (95%) and cognitive impairment (90%) in our cohort were observed to reflect the results of Schrag et al., who demonstrated the profound impact of these symptoms on patients [20]. Interestingly, sexual dysfunction was reported by only 3.33% of our cohort, a rate significantly lower than in other studies. This discrepancy could be attributed to cultural factors, as sexual dysfunction is often underreported in conservative societies, particularly in India. Such issues are less openly discussed due to cultural stigmas, which may lead to underreporting during clinical assessments. In contrast, studies conducted in more open societies often report higher prevalence rates for sexual dysfunction among PD patients.

Constipation was the most frequently reported GI symptom in our study, affecting 70% of patients, a finding consistent with global data (40-70%) and Indian studies. Factors like a vegetarian diet and lower fiber intake in India may exacerbate this symptom. Nausea or stomach sickness was reported by 40% of patients, often linked to delayed gastric emptying in PD. Indian studies on nausea in PD are limited, but international research connects it to autonomic dysfunction [21]. Less common symptoms like dysphagia (10%) and sialorrhea (9%) also contribute to the GI burden. Dysphagia can lead to severe complications, including aspiration pneumonia [22]. Sialorrhea, associated with reduced swallowing frequency, increases aspiration risk and requires focused management in Indian patients [23].

The majority of our patients (40 out of 60) had the tremor-predominant subtype, and these patients exhibited higher levels of pain, depression, and cognitive dysfunction. This finding is supported by Eggers et al., who observed similar symptom profiles in tremor-predominant PD patients [24]. Meanwhile, the akinetic-rigid subtype was associated with more severe GI issues and orthostatic hypotension.

In a further correlation study, it was found that depression and anxiety were among the most highly correlated NMS. This finding was corroborated by the work of Chaudhuri et al., who reported that mood disorders in PD were often interconnected and frequently appeared together [25]. Martinez-Martin et al. further noted that mood disturbances were found to significantly impair both motor and non-motor functions, contributing to overall disability in PD patients [26].

Limitations and future direction

This study's limitations include a relatively small sample size, limiting generalizability, and a cross-sectional design, restricting insights into disease progression. Including age- and gender-matched control groups would enhance comparative analyses. Future research should incorporate larger, multicenter, longitudinal studies to better understand NMS evolution and its cumulative impact on quality of life. Additionally, developing predictive models based on NMS severity could help identify patients at higher risk for reduced quality of life, facilitating early and targeted interventions tailored to patient age and PD subtype.

## Conclusions

This Indian study demonstrates that NMS, such as GI issues, depression, anxiety, pain, and cognitive impairment, are highly prevalent in PD patients. The more severe symptoms included GI issues, pain, and depression; however, symptoms like sexual dysfunction and psychosis were less severe. Emotional symptoms tend to occur more in the younger population, while physical symptoms tend to increase in the elderly. The tremor-predominant patients exhibited a higher prevalence of depression, pain, and cognitive issues, while the akinetic-rigid patients had increased incidences of GI symptoms and orthostatic hypotension. Insights from this study suggest that patients with PD require tailored approaches to symptom management as per their age group and clinical subtypes, which is a growing area of interest in clinical PD research. The therapeutic interventions to target both motor and NMS simultaneously may be more effective in reducing the overall burden of the disease. A more holistic, multidisciplinary approach to PD treatment, addressing both motor and NMS, is recommended for improving quality of life. Future research should prioritize understanding the progression of NMS and their cumulative effects on quality of life through larger studies.
